# Immune System–Tumor Crosstalk Under Microgravity: Mechanistic Insights, Challenges, and Translational Perspectives

**DOI:** 10.3390/cancers17172737

**Published:** 2025-08-23

**Authors:** Seyedesomaye Jasemi, Elena Rita Simula, Yao Lin, Rosanna Rita Satta, Corrado Rubino, Antonio Cossu, Milena Fais, Marta Noli, Leonardo A. Sechi

**Affiliations:** 1 Department of Biomedical Sciences, Division of Microbiology and Virology, University of Sassari, 07100 Sassari, Italy; ersimula@uniss.it (E.R.S.); linyao1163@163.com (Y.L.); faismilena@gmail.com (M.F.); martanoli@outlook.it (M.N.); 2 Department of Medicine and Pharmacy, University of Sassari, 07100 Sassari, Italy; rrsatta@uniss.it (R.R.S.); corubino@uniss.it (C.R.); cossu@uniss.it (A.C.); 3 Struttura Complessa Microbiologia e Virologia, Azienda Ospedaliera Universitaria Sassari, 07100 Sassari, Italy

**Keywords:** microgravity, immune system, cancer, immunoediting, immune–tumor interaction, human endogenous retroviruses (HERVs)

## Abstract

Despite notable progress in cancer therapy, immune evasion remains a major obstacle to effective treatment outcomes. In the context of spaceflight, astronauts are exposed to unique environmental stressors—particularly microgravity and radiation—that profoundly affect cellular and immune homeostasis. Emerging evidence suggests that microgravity alters key cellular processes, including proliferation, apoptosis, adhesion, and oncogenic signaling pathways such as NF-κB and ERK1/2. Concurrently, microgravity (µg) disrupts immune regulation, potentially facilitating both tumor progression and treatment resistance. Of particular concern is the upregulation of human endogenous retroviruses (HERVs), especially HERV-K and HERV-W, under µg conditions, which may exacerbate inflammatory responses and immune system dysregulation. While some studies indicate that µg may impair tumor growth, others reveal enhanced immune evasion and reduced antitumor immunity. Importantly, insights from µg research extend beyond space medicine and provide translational opportunities for terrestrial oncology, including the development of physiologically relevant 3D tumor models for drug screening, the identification of mechano-sensitive pathways (FAK/RhoA, YAP/TAZ) as therapeutic targets, and novel immunotherapeutic strategies involving epigenetic modulation and checkpoint inhibition. This review critically examines the dual role of µg in modulating cancer progression and immune function. We synthesize findings on how µg shapes immune responses, alters tumor–immune system interactions, and impacts the efficacy of immunotherapeutic approaches. Finally, we highlight translational opportunities and challenges for optimizing cancer immunotherapy and precision oncology in both spaceflight and Earth-based environments.

## Simple Summary

Cancer remains one of the leading causes of death, and overcoming the ability of tumors to escape the immune system is a major challenge. Astronauts in space are exposed to unique conditions, such as microgravity, which can change how cells and the immune system work. These changes may influence how tumors grow and respond to treatment. In this review, we explore how microgravity can both slow down and promote cancer, affect immune defenses, and alter communication between tumors and immune cells. We also discuss how this knowledge can be used on Earth to improve cancer treatment, including the design of more realistic laboratory models, the discovery of new drug targets, and the development of better immunotherapies. Our goal is to translate insights from microgravity research into strategies that can advance cancer treatment on Earth.

## 1. Introduction

Cancer is the second leading cause of death worldwide, accounting for over 13% of all human fatalities [1]. This complex disease develops through the gradual accumulation of genetic and epigenetic alterations, which reflect the underlying biological dysregulation driving cells toward neoplastic transformation [2,3]. These well-characterized hallmarks of cancer include uncontrolled proliferation, resistance to apoptosis, genomic instability, evasion of immune surveillance, induction of angiogenesis, and metastasis to distant organs [4]. Such molecular alterations are often driven by dysfunctions in key intracellular signaling pathways, such as PI3K/AKT, RAS/RAF/MEK/ERK, and Wnt/β-catenin, which are crucial in maintaining the balance between cell growth, survival, and differentiation [5,6]. Ultimately, cancer arises when the balance that normally controls cell growth, differentiation, and death is disrupted by complex interactions between internal cellular changes and signals from the surrounding immune and inflammatory environment [7].

Meanwhile, the immune system plays a dual and delicate role in the control and progression of cancer [8]. While the immune system enables the detection and elimination of abnormal cells, prolonged inflammation or immune dysfunction can impair this process, allowing tumors to grow and escape immune control [7,8]. The concept of cancer immunoediting captures this fragile balance by describing three phases: elimination, equilibrium, and escape [9]. It reflects how the immune system may initially eliminate cancer cells, then keep them in check for some time, but will eventually lose control as the disease progresses [9].

Cancer immunotherapy, by harnessing the immune system’s natural ability to recognize and eliminate malignant cells, has revolutionized cancer treatment. Strategies such as immune checkpoint inhibitors (e.g., PD-1/PD-L1 and CTLA-4 inhibitors), cytokine-based therapies (e.g., IL-2), tumor vaccines, and chimeric antigen receptor T (CAR-T) cells represent major advancements in this field and are increasingly being used in the treatment of various cancers [10,11,12].

In recent years, investigating the effects of space-related conditions—particularly microgravity (µg)—on immune function and tumor biology has become a rapidly growing and interdisciplinary field in biomedical research [13,14]. µg refers to the near-weightlessness experienced in space stations, where gravitational forces are significantly reduced but not entirely absent [15]. Considering the inherent limitations of space studies—including high cost, limited mission time, and interference factors such as radiation—several platforms have been developed to simulate microgravity (simulated microgravity, s-µg) on Earth. These include devices like the clinostat, random positioning machine (RPM), rotating wall vessel (RWV), Rotary Cell Culture Systems (RCCS), parabolic flight, and free-fall towers, all of which allow for controlled and reproducible studies by reducing or eliminating the gravitational vector [16].

Both in-flight and ground-based studies using astronaut samples, cell cultures, and animal models have demonstrated that spaceflight and s-µg can induce structural and molecular alterations across diverse cell types [17,18,19]. These changes contribute to a range of disorders, including skeletal muscle atrophy, vascular and cardiovascular dysfunctions, and immune system dysregulation [20,21,22]. Among affected systems, the immune system—an essential network for host defense—has consistently been shown to be one of the most affected physiological systems under µg conditions [23]. Changes and imbalances in the immune system have been linked to various disease conditions, including infectious diseases, neuroinflammation, aging, and cancer [24].

Interestingly, µg affects not only immune function but also tumor dynamics [13]. An expanding body of evidence shows that both real and s-µg influence the behavior of various cancer cell types (such as thyroid, breast, brain, and prostate), altering cell morphology, transcriptomic profiles, and signaling pathways like NF-κB, PI3K/AKT, ERK1/2, and YAP/TAZ [25,26]. Moreover, preliminary findings suggest that µg may induce epigenetic stress, potentially activating human endogenous retroviruses (HERVs) [19,27,28], adding an additional layer of complexity to tumor–immune interactions.

In this review, we aim to explore how µg influences the immune–tumor interplay, with a focus on immune regulation, tumor behavior, and implications for cancer therapy in space environments.

## 2. The Immunoediting Process in Cancer: Elimination, Equilibrium, and Escape

The human immune system is a complex and coordinated network responsible for maintaining homeostasis, defending against pathogens, and identifying cells undergoing neoplastic transformation [29]. This system consists of two main arms: innate immunity and adaptive immunity. Innate immunity, as the body’s first line of defense, initiates rapid, nonspecific, and effective responses to environmental threats. These responses are mediated by cells such as neutrophils, monocytes, macrophages, and natural killer (NK) cells, as well as soluble molecules including cytokines and complement proteins [29]. In contrast, adaptive immunity is a specific, delayed, and memory-based response mediated by T and B lymphocytes [29].

Immune system dysfunction and immune–inflammatory imbalances are closely associated with multiple stages of cancer development and progression, and are recognized as some of the key features of tumorigenesis [4]. Accumulating evidence indicates that the immune system plays a dual role in cancer: in early stages, acute and effective immune responses can prevent neoplastic growth by identifying and eliminating transformed cells; however, if inflammation persists or regulatory mechanisms become impaired—particularly under chronic inflammatory conditions—these same responses may promote angiogenesis, facilitate immune escape, and support the emergence of more aggressive tumor phenotypes [30].

**Figure 1:**
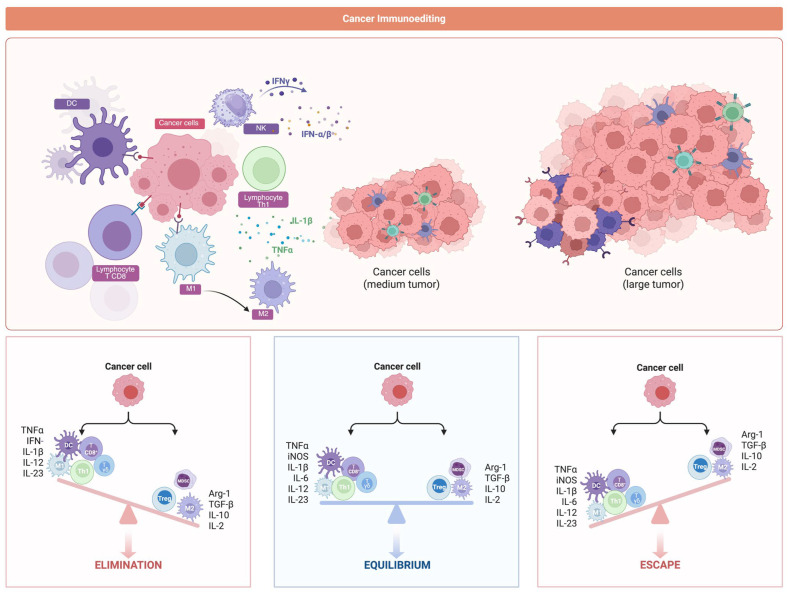
The three phases of cancer immunoediting: elimination, equilibrium, and escape. This diagram illustrates the dynamic interaction between tumor cells and the immune system. In the elimination phase, immune effector cells (NK cells, CD8^+^ T cells, Th1 cells, and M1 macrophages) dominate, supported by pro-inflammatory cytokines such as IFN-γ, TNF-α, IL-1β, IL-12, and IL-23, facilitating tumor recognition and destruction. The equilibrium phase reflects a state of immune–tumor standoff, characterized by a balance between inflammatory and immunosuppressive signals, including IL-6 and IL-17 alongside TGF-β and IL-10. In the escape phase, tumor-promoting immune subsets (M2 macrophages, regulatory T cells, and MDSCs) predominate, and the secretion of inhibitory cytokines (notably TGF-β, IL-10, and IL-2) suppresses effective antitumor immunity, allowing for tumor progression and immune evasion.

Based on these observations, the concept of cancer immunoediting has been introduced as a central framework describing the dynamic interaction between tumors and the immune system, consisting of three successive phases: elimination, equilibrium, and escape (Figure 1) [30,31].

During the elimination phase, the immune system—particularly the innate arm—initiates a coordinated antitumor response by recognizing tumor-associated antigens (TAAs) and tumor-specific antigens (TSAs) through pattern recognition receptors (PRRs) on dendritic cells (DCs) and M1 macrophages [31,32]. Activation of signaling pathways such as TLR–MyD88 and cGAS–STING–TBK1–IRF3 leads to the induction of key transcription factors NF-κB and IRF3, resulting in the secretion of pro-inflammatory cytokines such as IFN-α/β, IL-12, and TNF-α. M1 macrophages play a central role by releasing IL-12 and TNF-α, promoting Th1 polarization and apoptotic signaling in tumor cells [32,33]. NK cells contribute by recognizing MHC class I-deficient cells via activating receptors like NKG2D, NKp30, and DNAM-1, and triggering cytotoxic pathways involving PI3K–Akt and MAPK signaling [32]. IFN-γ secretion by NK cells further supports Th1-skewing, enhances CD8^+^ T cell cytotoxicity, and activates type 1 DCs.

γδ T cells, characterized by MHC-independent recognition and limited TCR diversity, also contribute to early immune defense through the secretion of TNF-α, IFN-γ, and granzymes. However, their role is context-dependent and can vary between anti-tumor and pro-tumor activities depending on cytokine milieu [32,33].

In the adaptive arm, CD8^+^ T cells differentiate into cytotoxic T lymphocytes (CTLs) following the recognition of tumor antigens via MHC-I and co-stimulatory signals like CD28. These CTLs eliminate target cells through granzyme B, perforin, Fas–FasL, and TRAIL–DR5 pathways. Concurrently, CD4^+^ T cells differentiate into Th1 subsets and secrete IFN-γ, amplifying CTL responses and reinforcing DC activation. Mature DCs, by upregulating MHC molecules and co-stimulatory proteins such as B7-1 and B7-2, play pivotal roles in antigen presentation and memory T cell priming [5]. Together, the elimination phase represents a coordinated and multi-layered immune assault on early neoplastic lesions, although efficiency varies depending on tumor type, mutation burden, and immune context [31,34].

During the equilibrium phase, tumor cells that have escaped initial immune destruction are kept under selective pressure by a weakened but persistent immune response. This phase is characterized by chronic low-grade inflammation, functional lymphocyte exhaustion, and the accumulation of immunosuppressive cytokines such as IL-10, TGF-β, and IL-35, which collectively shift the immune response from a cytotoxic to regulatory phenotype [31,34].

Macrophages undergo polarization from M1 to M2 or tumor-associated macrophage (TAM) phenotype, driven by signaling pathways including IL-4Rα, PI3K/Akt, and STAT6. These M2/TAM cells secrete pro-tumorigenic mediators such as VEGF, MMPs, and CSF-1, contributing to immune suppression, angiogenesis, and remodeling of the tumor microenvironment [35].

Regulatory T cells (Tregs), marked by FOXP3 expression and activated via the IL-2–STAT5 axis, suppress antitumor immunity by inhibiting DC maturation, reducing CTL activity, and secreting inhibitory cytokines. Concurrently, tolerogenic DCs—modulated by IL-10 and TGF-β—fail to present tumor antigens effectively and may promote the expansion of Tregs. Moreover, upregulation of immune checkpoint molecules such as PD-1, CTLA-4, TIM-3, and LAG-3 on T cells leads to diminished co-stimulatory signaling (e.g., via CD28), reduced cytokine production, impaired proliferation, and the induction of T cell exhaustion. This sustained checkpoint signaling, and antigen-presentation dysfunction represent pivotal immune escape strategies [36].

In the immune escape phase, tumor cells successfully evade immune surveillance through mechanisms such as downregulation of TAAs, loss or reduced MHC-I expression, activation of epithelial–mesenchymal transition (EMT), metabolic reprogramming via HIF-1α, and increased lactate production, which contribute to immune suppression and tumor progression [31,34,36].

At this stage, immunosuppressive components of the tumor immune microenvironment (TIME) reach peak activity. Cells such as M2-polarized TAMs, Tregs, and myeloid-derived suppressor cells (MDSCs) dominate, dampening antitumor immunity. MDSCs, activated through STAT3, NF-κB, and COX-2 pathways, exert potent immunosuppressive effects by producing Arginase-1, iNOS, NO, and ROS. These molecules inhibit CD8^+^ T cells and NK cells and promote immune evasion via the upregulation of PD-L1 and IDO [34,35,36,37].

Tumor-derived macrovesicles and exosomes act as vehicles for immunosuppressive cytokines and immune-checkpoint ligands, further shaping a tolerant microenvironment. Their release correlates with T cell death and checkpoint upregulation, reinforcing immune escape [38]. The persistence of chronic inflammation and immunosuppression culminates in T cell dysfunction, loss of immunological memory, and sustained immune evasion [34,36]. Notably, NF-κB functions as a central regulatory hub across these phases: while its activation during early immune responses promotes pro-inflammatory cytokine production and antitumor activity, persistent or dysregulated NF-κB signaling in chronic inflammation and within MDSCs contributes to immune suppression, checkpoint upregulation, and tumor immune escape [7].

Targeting these critical dysfunctions offers promise for enhancing immunotherapy efficacy. While the escape phase reflects immune failure, it also reveals therapeutic targets for restoring antitumor immunity. Understanding immune escape mechanisms informs clinical interventions and provides insight into how these processes might be altered under specific physiological conditions such as µg [34,36].

## 3. Microgravity and Immune System Dysregulation

µg is increasingly recognized as a powerful environmental stressor that profoundly impacts the immune system [23]. Over the past four decades, both real and s-µg have been shown to compromise immune cell development, differentiation, and function, leading to dysregulated immune surveillance and impaired anti-tumor responses [17,27,39].

Short-term missions or prolonged spaceflights can substantially impair immune cell function, particularly affecting T lymphocytes and NK cells [40,41]. While initial observations primarily attributed these changes to physical, psychological, and nutritional stressors inherent to space travel, emerging evidence indicates that µg itself functions as an independent physiological modulator of immune homeostasis [42].

Both flight-based datasets and in vitro studies have revealed marked immunophenotypic alterations following µg exposure, even in the absence of classical stress markers. These include a significant reduction in NK cell counts, increased proportions of monocytes and B cells, and altered expression of adhesion molecules such as CD62L, potentially linked to cytoskeletal remodeling or transcriptional regulation pathways like NF-κB [40]. Among these regulatory shifts, suppression of NF-κB signaling has been repeatedly observed in immune cells under µg [43,44].

Such changes may impair lymphocyte migration, differentiation, and crosstalk with other immune cells.

Evidence from both human and animal studies has demonstrated increased mitochondrial damage, elevated ROS production, and diminished antioxidant capacity in immune cells under µg [45,46]. These alterations may predispose individuals to chronic inflammation, immune exhaustion, and functional lymphocyte impairment [47].

Clinically, these immune disturbances correlate with a higher incidence of opportunistic infections and inflammatory complications in astronauts. Reports of latent virus reactivation—such as Epstein–Barr virus (EBV), cytomegalovirus (CMV), and varicella-zoster virus (VZV)—as well as allergic responses and atopic dermatitis, have been documented during extended missions [48,49,50].

Importantly, multi-omic datasets from the recent SOMA program and NASA Twins Study provide strong molecular evidence for these clinical observations [51,52]. Single-cell and cfRNA analyses revealed a conserved “spaceflight signature” characterized by enrichment of oxidative phosphorylation and immune-regulatory pathways. Notably, T cells exhibited FOXP3 upregulation, indicative of increased Treg differentiation, while MHC class I genes were persistently suppressed, suggesting compromised antigen presentation. Epigenetic remodeling was also evident, including promoter hypermethylation in NOTCH3 and SLC1A5—key regulators of T-cell differentiation and activation—accompanied by reduced IL-2 signaling [51,52]. These changes coincided with systemic inflammatory signatures marked by elevated IL-6, TNF-α, and VEGF, and a Th1-to-Th2 polarization shift. Collectively, these findings highlight a µg-driven immune profile that mimics hallmarks of tumor-associated immunosuppression, reinforcing concerns about impaired immunosurveillance during spaceflight and its relevance to cancer risk.

### 3.1. The Effect of Microgravity on the Innate Immune System

#### 3.1.1. Natural Killer Cells Under Microgravity

NK cells, as key effectors of the innate immune system, are essential for the rapid identification and elimination of virus-infected and malignant cells [29]. However, accumulating evidence suggests that both real and s-µg conditions impair the antitumor functionality of these cells [53].

In vitro studies using s-µg models have shown that short-term exposure (24–48 h) of human NK cells leads to a marked decrease in their cytotoxic activity against tumor cell lines such as K-562 and MOLT-4. This functional decline is accompanied by reduced expression of critical effector molecules, including IFN-γ, perforin, and, in some cases, granzyme B [54]. Additionally, increased rates of apoptosis have been observed under these conditions [54].

Moreover, µg exposure significantly downregulates the expression of activation markers such as CD25 and CD69, reflecting a weakened activation state and reduced responsiveness of NK cells [55]. Transcriptomic analyses further support these findings, revealing that gene expression profiles associated with cytotoxicity, migration, and activation pathways are broadly suppressed [55]. In particular, the diminished expression of activating receptors such as NKG2D and NKG2A underscores the compromised ability of NK cells to recognize and target tumor cells under µg [53].

A 2025 systematic review of 140 studies investigating isolation, confinement, and environmental (ICE) stressors confirmed previous observations of microgravity-induced immune dysfunction. A consistent decline in NK cell counts and reduced secretion of key antitumor cytokines—such as IFN-γ and IL-2—emerged as robust markers of immune suppression under µg conditions. Although some studies noted increases in RANTES (CCL5) and granulocyte levels, these findings were considered secondary and inconclusive, requiring further validation [41].

In ground-based animal models, such as hindlimb unloading, decreases in NK cells, B cells, and erythroid progenitors within the bone marrow were observed, along with an increase in hematopoietic stem cells (HSCs) and T lymphocytes [56]. These alterations suggest a shift in hematopoietic lineage commitment and a disruption of immune homeostasis. Notably, the delayed reconstitution of NK cells upon return to normal gravity raises the possibility of lasting functional or epigenetic modifications in HSCs.

Concordantly, analyses of blood samples from astronauts’ post-mission have revealed persistent alterations in the populations and functional capacities of NK cells, B cells, and HSCs [27]. This sustained dysregulation underscores the critical need to explore the long-term immunological consequences of spaceflight exposure.

Findings from classic animal spaceflight studies further support this trend. For example, mice aboard the COSMOS 2044 mission exhibited reduced NK cell-mediated lysis of mouse lymphoma cell line YAC-1 cells, although their response to human leukemia cell line K-562 targets was partially preserved [57]. Similarly, astronauts on the International Space Station (ISS) displayed an approximately 50% decline in NK cell cytotoxicity against K-562 cells by day 90 of the mission [58]. This impairment was more pronounced among first-time flyers and occurred despite stable NK cell counts and unaltered levels of perforin and granzyme B, implying disruptions in functional or metabolic pathways [58].

#### 3.1.2. Macrophages Under Microgravity

Macrophages, as central players in the innate immune system, are crucial for the recognition, phagocytosis, and elimination of pathogens and tumor cells, as well as for orchestrating the immune microenvironment [29]. Emerging evidence indicates that exposure to µg—whether in simulated platforms such as RWV bioreactors and parabolic flights, or in real space missions—induces profound structural and functional changes in these cells [59,60,61].

One of the earliest and most significant effects is observed at the level of the macrophage cytoskeleton. µg disrupts the organization of essential cytoskeletal elements, including actin filaments, tubulin, and the Arp2/3 complex [62]. This disorganization impairs macrophage motility, adhesion, and the formation of cellular protrusions, ultimately compromising their ability to migrate to the sites of inflammation or tumors and to effectively interact with T lymphocytes.

Functionally, µg markedly affects macrophage polarization. Under these conditions, macrophages often fail to undergo complete differentiation into classical M1 or alternative M2 phenotypes or may exhibit mixed phenotypes with contradictory features [44,63]. Notably, many studies report a shift toward an M2-like phenotype, characterized by an increased expression of IL-10 and VEGF, along with decreased TNF-α levels—features indicative of immunosuppressive reprogramming and a tumor-permissive immune profile [44].

At the molecular level, critical signaling pathways involved in macrophage activation and immune regulation—including RAS/ERK, NF-κB, MAPK, and HIF-1α—are significantly downregulated under µg [43,64]. NF-κB suppression, in particular, acts as a pivotal checkpoint by diminishing the transcription of pro-inflammatory cytokines (e.g., TNF-α, IL-6), reducing costimulatory molecule expression, and promoting an immunosuppressive macrophage phenotype [43]. Conversely, the p38-MAPK → C/EBPβ → Arginase I axis is upregulated, promoting Arginase I expression [64]. This shift limits arginine availability and suppresses nitric oxide synthesis, both of which contribute to diminished antitumor responses and enhanced immune tolerance in the µg environment.

A large-scale study conducted in 2016, which analyzed over 8000 molecular pathways in human cells exposed to µg, revealed a significant downregulation of immune and inflammatory responses [65]. Among the key mechanisms identified was the inhibition of NF-κB signaling through the Notch1 pathway, suggesting a central role in immune suppression. Concurrently, activation of cancer-related pathways, including those associated with leukemia and liver cancer, was observed, along with enhanced cellular sensitivity to anticancer drugs [65].

µg also impairs classical macrophage functions such as phagocytosis, ROS production, IL-1 secretion, and the expression of adhesion molecules including ICAM-1, CD14, and CD18 [66]. These impairments collectively diminish the capacity of macrophages to recognize pathogens, initiate immune responses, and effectively interact with T and NK cells [66].

Nevertheless, cytokine production by macrophages under µg appears to be variable and, in some cases, contradictory. Studies have reported that the direction and magnitude of cytokine responses—including IL-1, IL-6, IL-8, and TNF-α—are influenced by factors such as cell type (primary versus cell lines), the intensity of simulated gravity, and exposure duration. Some experiments demonstrated increased expression of inflammatory cytokines [67,68], while others reported suppression [69].

Overall, current evidence suggests that µg not only alters the structural organization of macrophages but also fundamentally reprograms their functionality, differentiation trajectory, and intracellular signaling networks. This immune reprogramming, characterized by a shift toward immunosuppressive phenotypes, may weaken macrophage-mediated antitumor responses and potentially increase the risk of cancer development or progression under spaceflight conditions. Therefore, precise characterization of these changes and the implementation of immunomodulatory strategies to preserve or restore macrophage function are essential in the context of long-duration space missions and space-based immunotherapies.

#### 3.1.3. Dendritic Cells Under Microgravity

DCs, as essential mediators between innate and adaptive immunity, exhibit a biphasic response to s-µg conditions. Short-term exposure (up to 3 days) has been associated with the activation of intracellular signaling pathways including phosphorylated STAT5, ERK1/2, and mTOR, alongside increased expression of maturation markers such as MHC-I/II, CD80, and CD86—features indicative of DC activation and early maturation. In contrast, prolonged exposure (4 to 14 days) results in a marked decline in these indicators and a reduced capacity of DCs to stimulate T lymphocytes [70]. This transition likely reflects an adaptive or tolerogenic shift, accompanied by decreased IL-2 expression, suggesting functional exhaustion or immunogenic suppression [70].

Further studies have demonstrated that extended s-µg exposure inhibits the NF-κB pathway and downregulates the expression of critical costimulatory molecules, including MHC-II, CD80, and CD86 [70,71]. These impairments significantly compromise DC-mediated CD4^+^ T cell activation, survival, and polarization toward effective subsets such as Th1 and Th17 [72]. Such dysfunctions in DC biology represent a key mechanism contributing to the attenuation of adaptive immunity and may help explain the increased susceptibility of astronauts to infections and cancer [70].

Similarly, γδ T cells—bridging innate and adaptive responses—are functionally impaired under s-μg. A reduction in their numbers, decreased production of IFN-γ and TNF-α, and diminished cytotoxicity have all been observed. In parallel, the increase in regulatory T cells (Tregs), along with the impaired function of DCs and cytotoxic T lymphocytes (CTLs), collectively contributes to a broad immunosuppressive state that favors immune tolerance and may facilitate tumor immune escape [3].

Recent in vivo findings have corroborated these observations. In a zebrafish model exposed to simulated μg, Zhu et al. demonstrated significant downregulation of antiviral signaling pathways, including RIG-I-like receptors (RLRs) and Toll-like receptors (TLRs) [73]. Notably, μg disrupted TRIM25-mediated ubiquitination of RIG-I, thereby impairing a key amplification loop in antiviral immunity [73]. As these pathways are also involved in tumor immunosurveillance [9], their suppression under µg may promote both viral reactivation and increased cancer risk.

### 3.2. The Effect of Microgravity on the Adaptive Immune System

#### 3.2.1. CD8^+^ Cytotoxic T Cells Under Microgravity

µg, as a key environmental stressor in spaceflight, exerts profound effects on the adaptive immune system, particularly on CD4^+^ and CD8^+^ T lymphocytes—both central to antitumor immunity [74]. Robust evidence from in vitro experiments, animal models, and human transcriptomic analyses indicates that µg not only alters T cell function but also disrupts fundamental signaling, metabolic, and epigenetic pathways, thereby mimicking features of immune-privileged tumor microenvironments.

In CD8^+^ cytotoxic T cells, s-µg impairs effector functionality by reducing the number of IFN-γ-producing cells and attenuating degranulation, indicating a diminished cytotoxic capacity. These transcriptional and functional alterations compromise the immune system’s ability to eliminate malignant cells, as demonstrated by single-cell RNA-seq analyses of PBMCs and in-flight validation studies [27].

Moreover, decreased expression of activation markers including CD25 and CD69 reflects impaired proliferation and functional competence [75]. Inhibition of the JAK/STAT signaling cascade—particularly STAT1 and STAT5—has been confirmed in single-cell datasets and is linked to reduced differentiation and survival of CD8^+^ cells [27].

S-µg platforms such as clinostats and RCCS have shown that even brief exposures (5–60 min) can diminish expression of T cell receptor (TCR)-associated molecules like CD3, ZAP70, and IL-2 receptor [18]. These changes persist even under anti-CD3 stimulation, pointing to early disruption of TCR signaling. Mechanistically, such impairments are likely due to cytoskeletal disorganization and disturbed TCR-MHC interactions, which may broadly compromise immunological memory and adaptive immune responses.

#### 3.2.2. CD4^+^ T Cells and Treg Under Microgravity

µg also influences CD4^+^ T cell polarization, favoring differentiation toward Th2 and regulatory Treg phenotypes over Th1 responses. This immunological skewing is associated with increased FOXP3 expression, elevated IL-10 and TGF-β levels, and upregulation of checkpoint molecules such as CTLA-4, reflecting a shift toward an immunosuppressive and potentially exhausted T cell state [76]. CyTOF-based studies further reveal an increase in activated CD4^+^ T cell populations and an expanded Treg response during prolonged exposure, characterized by elevated STAT5 phosphorylation, CTLA-4 expression, and a lack of CD25—features commonly associated with T cell exhaustion, senescence, and impaired tumor surveillance [27]. In addition, µg disrupts mitochondrial function in T lymphocytes, leading to decreased ATP production, increased electron leakage, elevated ROS levels, and loss of mitochondrial membrane potential [77,78].

#### 3.2.3. B Lymphocytes Under Microgravity

B lymphocytes are essential for humoral immunity, yet their response to µg has been less studied compared to T cells. Evidence from animal models indicates that µg can reduce B-cell populations and alter hematopoiesis. In mice exposed to simulated µg through hindlimb unloading, the percentage of B lymphocytes in both bone marrow and spleen declined significantly, accompanied by changes in hematopoietic regulatory genes such as Flt3L, GM-CSF, and IL-3, as well as cytoskeletal disruption in stromal cells [79]. Rodent spaceflight studies—including missions like the BION-M1 biosatellite—have demonstrated a significant alteration in splenic B-cell numbers after approximately one month onboard [80]. In contrast, data from human analog or orbital missions, such as two-month head-down bed rest studies and several ISS missions, reported no significant alterations in B-cell subsets or immunoglobulin levels, suggesting that species differences, mission duration, or countermeasures may moderate the impact on humoral immunity [81].

Mechanistic studies provide further insight into how µg affects B-cell viability and function. In vitro experiments using human B lymphoblastoid cells (HMy2.CIR) demonstrated that simulated µg can amplify stress responses when combined with other space-relevant factors such as radiation. Specifically, µg enhanced heavy-ion radiation-induced apoptosis by increasing reactive oxygen species (ROS) production and activating the ERK/MKP-1/caspase-3 pathway; these effects were significantly reduced by antioxidant treatment with N-acetyl cysteine or quercetin [82]. Complementary transcriptomic analyses have also revealed reprogramming of genes related to B-cell differentiation and chromatin organization under combined µg and low-dose radiation, even without major changes in cell counts [83]. Collectively, these findings suggest that µg can compromise humoral immunity through oxidative stress, hematopoietic disruption, and molecular reprogramming, highlighting the need for more in-depth studies and countermeasures to protect astronaut health during long missions.

#### 3.2.4. Transcriptomic and Pathway Reprogramming: Implications for Cancer

Multiple keys signaling pathways in adaptive immune cells—including NF-κB, MAPK, JAK/STAT, HIF-1α, and RAS/ERK—are significantly suppressed under µg conditions. Inhibition of NF-κB results in reduced transcription of pro-inflammatory cytokines such as TNF-α and IL-1β, while suppression of MAPK signaling leads to diminished IFN-γ production [84,85]. Downregulation of the JAK1/STAT1 axis impairs the expression of interferon-responsive genes, weakening Th1-mediated immune responses [85]. Additionally, HIF-1α, a key gravitational sensor, is inversely regulated during parabolic and suborbital flight exposures, resulting in a dramatic reduction in its expression and that of its downstream effector, PDK1 [85].

Despite the prevailing evidence that µg suppresses adaptive immunity, a groundbreaking study by Bradley et al. (2019) challenged this paradigm by demonstrating that s-µg can paradoxically impair tumor immune evasion mechanisms [86].

In their murine model, E.G7 lymphoma cells, which typically suppress DC function to escape immune detection, lost this immunosuppressive capacity under s-µg conditions. As a result, CD4^+^ T cells exhibited increased IL-2 production, CD8^+^ T cells secreted more IFN-γ, and overall tumor growth was significantly reduced [65]. These findings highlight that under specific conditions, µg-induced cellular stress may enhance tumor immunogenicity, rendering malignant cells more susceptible to immune-mediated elimination.

Exposure to µg induces extensive reprogramming of molecular pathways involved in immune regulation and tumorigenesis. This shift can concurrently impair immune surveillance while enhancing tumor-promoting processes. Transcriptomic profiling and gene set enrichment analysis (GSEA) from spaceflight and ground-based simulation models have consistently revealed the upregulation of oncogenic pathways, including KRAS, MYC targets, mTORC1, E2F targets, and the Unfolded Protein Response (UPR) [84]. Conversely, critical immune-related signaling cascades such as TNFα/NF-κB, TCR signaling, NFAT, and Notch1 are functionally suppressed under these conditions [84].

**Figure 2:**
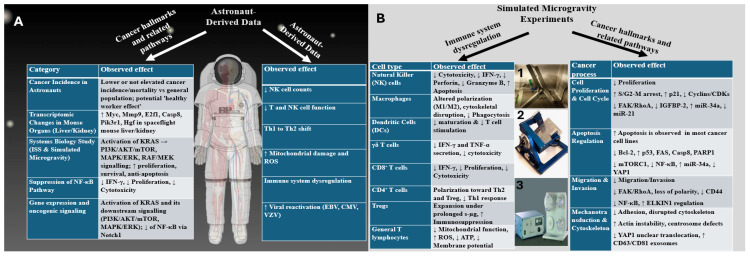
Effects of microgravity on immune dysregulation and cancer processes. The left panel (**A**) summarizes astronaut-derived data, while the right panel (**B**) presents findings from simulated microgravity (s-µg) experiments. Images illustrate commonly used s-µg platforms: (1) Clinostat: Rotates samples along a single axis to randomize the gravity vector; (2) Random Positioning Machine (RPM): Provides multidirectional rotation for 3D gravity vector randomization; (3) Rotating Wall Vessel (RWV)/Rotary Cell Culture System (RCCS): Simulates low-shear microgravity through horizontal rotation in a fluid-filled chamber, supporting 3D cell culture. Upregulation is indicated with upward arrows (↑), while downregulation is indicated with downward arrows (↓).

For instance, the mTORC1 pathway, which is pivotal for T cell metabolism, survival, and effector function, remains aberrantly activated under µg and may contribute to impaired memory T cell formation and an expansion of Tregs [87]. Chronic suppression of the NF-κB pathway—central to the production of inflammatory cytokines like TNF-α and IL-1β—has been widely reported across multiple spaceflight and s-µg models, correlating with reduced functionality of T cells, macrophages, and NK cells [87,88]. Additionally, inhibition of TCR and NFAT signaling has been shown to alter the activation and differentiation of both CD4^+^ and CD8^+^ T cells in murine, human, and in-flight models [87,88]. These molecular alterations disrupt the immune–tumor interface by impairing the recognition and elimination of malignant cells, while simultaneously supporting hallmarks of cancer, including proliferation, immune evasion, and therapy resistance. Notably, these changes have been observed even in the absence of confounding stressors such as ionizing radiation, highlighting that µg alone is sufficient to rewire cellular networks. These insights underscore the critical role of µg in modulating the immune–oncology axis and support the rationale for developing gravity-adapted immunotherapeutic strategies for space medicine and terrestrial applications (Figure 2).

### 3.3. Indirect Drivers of Immune Dysregulation Under Microgravity

#### 3.3.1. Neuroendocrine Stress and Immune Suppression

µg indirectly disrupts immune surveillance by altering neuroendocrine regulation, mainly through activation of the hypothalamic–pituitary–adrenal (HPA) axis and the sympathoadrenal system [89]. The absence of gravitational load acts as a strong physiological stressor, triggering increased hypothalamic release of corticotropin-releasing hormone (CRH), enhanced ACTH secretion from the pituitary, and a systemic rise in cortisol and catecholamines [89,90]. Cortisol binds to glucocorticoid receptors on lymphocytes, blocking NF-κB signaling and lowering IL-2 and IFN-γ transcription, which impairs T-cell proliferation and cytotoxicity [91]. Moreover, catecholamine-driven β_2_-adrenergic signaling raises intracellular cAMP and activates PKA, suppressing IL-12 production in dendritic cells and shifting CD4^+^ T cells toward a Th2 profile, weakening antitumor immunity [92,93]. NK-cell cytotoxicity is further reduced, while macrophages tend toward an M2-like phenotype, creating a tumor-supportive microenvironment [44,54,55]. Multi-omics data from space-exposed mouse brains also show epigenetic remodeling in hypothalamic and limbic regions, amplifying neuroendocrine stress and its immune system dysregulation [94]. Together, these mechanisms generate a systemic immunosuppressive state in microgravity, limiting cancer immunosurveillance and potentially reducing immunotherapy effectiveness.

#### 3.3.2. Stromal and Metabolic Reprogramming

µg strongly influences stromal structure and metabolic balance, indirectly altering immune regulation. The absence of normal mechanical loading disrupts extracellular matrix (ECM) organization and mechanotransduction pathways such as integrin–FAK and YAP/TAZ signaling, which are key to cytoskeletal tension and immune–stromal communication [60,95]. These changes hinder dendritic cell maturation and T-cell activation, while promoting an immunosuppressive tumor stroma. At the cellular level, primary human macrophages exposed to long-term µg show metabolic reprogramming without major cytoskeletal disruption, including reduced ICAM-1 expression and loss of cell-surface fucosylation, along with elevated free fucose in culture supernatants—alterations that weaken macrophage–T cell interactions and dampen innate immune responses [96]. Systemically, unloading impairs skeletal interoception and activates hypothalamic neuropeptide Y pathways, increasing sympathetic activity and driving metabolic shifts toward bone resorption and altered fat metabolism [97]. These changes overlap with stress-induced insulin resistance and mitochondrial dysfunction, resembling aging-like profiles and promoting chronic low-grade inflammation, or “inflammaging,” as documented in long-term missions [52,98]. This paradox—persistent inflammatory signaling combined with functional immunosuppression—not only fosters immune escape but may also reduce the effectiveness of immunotherapy. Understanding these systemic changes is essential for designing countermeasures that integrate neuroendocrine, stromal, and metabolic support to maintain immune health during deep-space missions.

#### 3.3.3. The Effect of Microgravity on Human Endogenous Retroviruses (HERVs)

One of the most intriguing aspects of immune dysregulation in µg is the reactivation of HERVs [19,27]. Single-cell analyses of peripheral blood mononuclear cells (PBMCs) exposed to short-term s-µg (25 h) have demonstrated a marked upregulation of HERV expression, accompanied by immune alterations such as dysregulated cytokine signaling, and functional impairment of T and NK cells [27]. Similarly, increased expression of HERVs has been reported during and after spaceflight, correlating with systemic immune dysregulation and heightened inflammation, as reflected by elevated levels of IL-6, C-reactive protein (CRP), and interferon-stimulated genes across both short- and long-duration missions. The NASA Twins Study further demonstrated that a year-long mission on the ISS induces epigenetic remodeling, transcriptional reprogramming of immune and oxidative stress pathways, and fluctuations in telomere length followed by an increase in critically short telomeres post-flight, which collectively suggest a genomic landscape permissive to oncogenesis [17,99]. These findings are consistent with our previous observations showing significant upregulation of HERV genes and pro-inflammatory cytokines (including IL-1, IL-6, and TNF-α) under s-µg in various cell types [19]. These observations suggest that µg creates a permissive environment for HERV activation, likely through epigenetic changes, oxidative stress, and immune dysregulation.

HERVs, which account for approximately 8% of the human genome, are remnants of ancient retroviral integrations. While controlled expression of these elements contributes to physiological processes such as placental development and immune regulation, their aberrant reactivation has been implicated in autoimmune disorders, neurodegeneration, and cancer [100,101,102,103,104]. Although direct in vivo evidence linking HERV activation in µg to tumorigenesis is lacking, mechanistic insights from terrestrial models provide a strong rationale to consider similar risks in spaceflight conditions.

One critical pathway involves the induction of a viral mimicry state, wherein derepressed HERV loci generate double-stranded RNAs (dsRNAs) resembling viral replication intermediates [105,106,107]. These dsRNAs serve as pathogen-associated molecular patterns (PAMPs) and are sensed by pattern recognition receptors (PRRs), including Toll-like receptor 3 (TLR3) in endosomes and melanoma differentiation-associated protein 5 (MDA5) in the cytosol [105,108]. PRR engagement activates antiviral signaling cascades through TRIF and mitochondrial antiviral signaling protein (MAVS), culminating in interferon regulatory factor (IRF) activation and type I/III interferon release (IFN-α/β and IFN-λ), accompanied by robust induction of interferon-stimulated genes (ISGs) [109,110,111]. These responses enhance antigen presentation via MHC-I and MHC-II upregulation, amplify cytokine secretion, and prime adaptive immunity, including CD8^+^ T-cell activation [110].

However, this defense-like response can become self-perpetuating. Interferon signaling, together with inflammatory transcription factors such as STAT1 and NF-κB, binds to interferon-stimulated response elements and NF-κB motifs within HERV long terminal repeats (LTRs), promoting chromatin opening and additional HERV transcription [112,113,114]. Such persistent activation raises concerns about immune exhaustion and tissue damage during long-duration missions.

Beyond innate immunity, HERV products function as tumor-associated antigens (TAAs), eliciting both humoral and cellular responses. Elevated HERV expression in multiple cancers correlates with increased immune infiltration and improved responsiveness to immune checkpoint blockade (ICB) therapies, including PD-1 and CTLA-4 inhibitors [115,116,117]. Epigenetic agents such as DNA methyltransferase inhibitors can derepress HERV loci, triggering an interferon response via the viral mimicry pathway and synergizing with immunotherapies [106,118]. HERV-derived peptides and envelope proteins have emerged as promising immunogenic targets for CAR-T cell therapies and cancer vaccines, with encouraging preclinical results [119,120,121]. These findings underscore the dual nature of HERV reactivation: while it can enhance tumor immunogenicity, it may also promote oncogenic processes under permissive conditions [122].

From an oncogenic perspective, epigenetic instability enables HERV LTRs to act as alternative promoters or enhancers for proto-oncogenes, a process termed onco-exaptation [112]. This mechanism is exemplified in Hodgkin’s lymphoma, where an ERV-derived LTR functions as an alternative promoter for the colony-stimulating factor 1 receptor (CSF1R) gene, driving its aberrant overexpression and subsequent activation of oncogenic signaling cascades such as PI3K/AKT and RAS/RAF/MEK/ERK pathways [123]. Similarly, in acute myeloid leukemia, CRISPR-mediated silencing of ERV-associated enhancers significantly impaired cell proliferation and survival, highlighting the pivotal role of ERVs in reprogramming cancer-related transcriptional networks [124].

**Figure 3:**
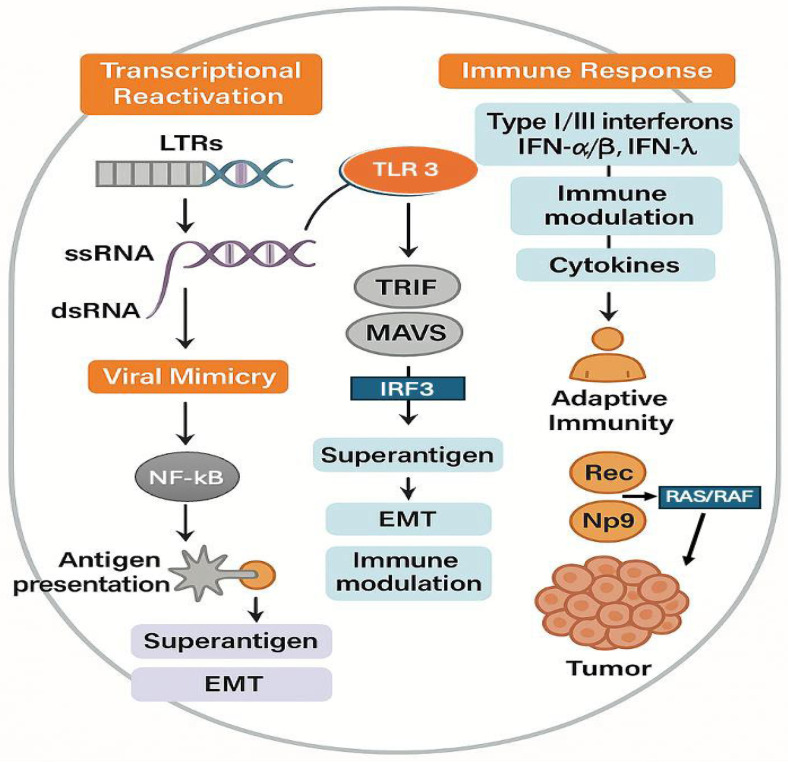
Proposed mechanistic model of HERV reactivation and immune–oncogenic effects under microgravity. Microgravity-induced epigenetic dysregulation may activate HERV long terminal repeats (LTRs), leading to transcription of viral RNAs and triggering viral mimicry pathways via TLR3–TRIF/MAVS signaling. This activates IRF3 and NF-κB, resulting in type I/III interferon production, cytokine release, antigen presentation, and adaptive immune responses. Persistent activation can amplify HERV expression and produce viral proteins such as Rec and Np9, which promote oncogenic signaling (e.g., RAS/RAF), epithelial–mesenchymal transition (EMT), and tumor progression. Model adapted from terrestrial studies, with limited but consistent spaceflight data supporting HERV upregulation in astronauts.

HERV-encoded proteins such as Env, Rec, and Np9 have been implicated in multiple oncogenic processes. Env facilitates abnormal cell–cell fusion and contributes to epithelial-to-mesenchymal transition (EMT) through the activation of signaling pathways including Wnt/β-catenin and TGF-β. Moreover, Rec and Np9 interact with key regulatory factors to activate oncogenic cascades such as the RAS/RAF/MEK/ERK and PI3K/AKT pathways, thereby promoting cellular transformation, metastatic dissemination, genomic instability, and resistance to therapy (Figure 3) [119,121,125,126].

Taken together, the convergence of µg-induced epigenetic remodeling, HERV reactivation, antiviral mimicry signaling, and oncogenic reprogramming delineates a plausible mechanistic link between spaceflight-associated immune dysregulation and cancer risk. Although direct evidence remains to be established in vivo, these interconnections justify targeted investigations to determine whether µg-driven HERV activation predominantly enhances immune surveillance or, paradoxically, facilitates immune escape and tumor progression. Addressing this uncertainty will be critical for risk mitigation and the design of combined epigenetic-immunotherapeutic strategies for astronauts and cancer patients alike.

## 4. Microgravity-Induced Tumor Cell Alterations

The National Aeronautics and Space Administration (NASA) has systematically identified and categorized a range of biological and medical risks associated with long-duration space missions. These risks are classified into three levels based on their severity: low-risk changes such as alterations in the microbiota; medium-risk changes such as telomere shortening; and high-risk changes that include genomic instability, neurovascular disorders, and immunosuppression [52]. Interestingly, many of these alterations—including telomere shortening, immune dysregulation, and genomic instability—are also well-known hallmarks of cancer biology [127]. The NASA Twins Study further demonstrated that a year-long mission on the ISS induces epigenetic remodeling, transcriptional reprogramming of immune and oxidative stress pathways, and fluctuations in telomere length followed by an increase in critically short telomeres post-flight, which collectively suggest a genomic landscape permissive to oncogenesis [52]. In addition, recent multi-omics studies from the SOMA program, including spatial transcriptomics of astronaut skin after short-duration missions, further reinforce these observations by revealing post-flight activation of KRAS signaling, epithelial barrier disruption, and pro-inflammatory responses, all of which are tightly linked to tumor initiation and progression pathways [48].

Despite these biological overlaps, epidemiological evidence to date has not conclusively demonstrated an increased cancer risk among astronauts. For instance, in a comprehensive cohort study analyzing data from American astronauts between 1959 and 2017, cancer incidence and mortality rates were not elevated and were even reported to be lower than in the general U.S. population [128]. These results may be attributed to strict medical selection criteria, healthier behaviors among astronauts, and the “healthy worker effect,” which favors a lower observed disease incidence in occupational cohorts [129].

Nevertheless, an increasing number of molecular studies have raised the possibility that µg may influence pathways implicated in cancer initiation and progression. For example, a study by Hammond et al. (2018) examined the transcriptomic profile of liver and kidney tissues in mice exposed to real µg during spaceflight, reporting increased expression of several cancer-related genes, including Myc, Mmp9, E2f1, Casp8, Pik3r1, and Hgf [130]. These genes play pivotal roles in key aspects of tumorigenesis, including regulation of cell proliferation, apoptosis, differentiation, and migration [65,131].

Building upon these findings, a systems biology study by Mukhopadhyay et al. (2016) employed transcriptomic analyses of human cells exposed to both real µg aboard the International Space Station (ISS) and s-µg on Earth [65]. The study found significant activation of the KRAS signaling pathway, which—through its downstream axes including PI3K/AKT/mTOR, MAPK/ERK, and RAF/MEK—promotes uncontrolled proliferation, survival under stress, apoptotic resistance, and enhanced invasiveness, all of which are characteristic of aggressive tumors [65,132]. Notably, these oncogenic signals were observed even in the absence of space radiation, suggesting that µg alone may be sufficient to induce neoplastic-like changes. In parallel, the study also reported a downregulation of the NF-κB pathway mediated by altered Notch1 signaling [65]. Given NF-κB’s pivotal role in regulating immunity, inflammation, and cell survival, its suppression may contribute to a permissive microenvironment for tumor development by impairing anti-tumor immune responses [133]. Taken together, these findings suggest that µg may directly influence fundamental processes such as apoptosis, proliferation, and migration—key hallmarks of cancer—which will be further explored in the following in vitro studies.

### 4.1. Disturbance in Cytoskeletal Structure, Mechanobiology, and Cellular Tensegrity in Microgravity

One of the earliest and most notable responses of cancer cells to µg conditions is the reorganization of the cytoskeleton and the disruption of the cellular tensegrity network [134]. Under normal gravity, the coordinated interaction between microtubules, actin microfilaments, and intermediate filaments not only maintains cell morphology but also supports the transmission of mechanical forces, activation of mechanosensitive signaling pathways, and regulation of key processes such as migration, proliferation, and apoptosis [135]. In contrast, μg disrupts this cytoskeletal network, compromising both its structural integrity and functional dynamics. Cancer cell lines such as A549, FTC-133 (follicular thyroid carcinoma), MDA-MB-231 (breast cancer), and H1703 (Squamous Cell Carcinoma) cell lines, when exposed to μg conditions, exhibit reduced F-actin density, altered filamentous architecture, and loss of focal adhesion organization—phenomena linked to diminished invasiveness, phenotypic reversion, and partial epithelial restoration.

In A549 cells, these alterations correlate with reduced cytoskeletal tension, inactivation of FAK and YAP/TAZ pathways, decreased adhesion and proliferation, and enhanced apoptosis [95]. The concept of cellular tensegrity—defined as the balance between tensile forces (e.g., actomyosin) and compressive forces (e.g., microtubules, ECM)—is profoundly affected under µg, leading to impaired mechano-transduction, disrupted ECM adhesion, and even alterations in nuclear morphology. Moreover, exposure to s-µg leads to alterations in nuclear morphology—including changes in surface-to-volume ratio—accompanied by a weakened association between Lamins A/C and the cytoskeleton. These mechanical disruptions are mediated via the LINC complex and have been linked to downstream epigenetic changes, including chromatin reorganization and altered gene expression [136,137].

µg-induced inhibition of the FAK/PI3K/AKT/mTOR and Hippo–YAP signaling cascades results in growth suppression, downregulation of anti-apoptotic genes, and EMT inhibition. Notably, YAP—a mechanosensitive transcriptional coactivator—has been shown to be suppressed under μg, leading to decreased expression of proliferation-associated genes and heightened sensitivity to chemotherapeutics such as cisplatin and doxorubicin.

Another hallmark response is the self-assembly of tumor cells into three-dimensional spheroid or pseudo-myeloid structures under µg [138,139]. These formations, arising from the removal of gravitational mechanical stress and cytoskeletal rearrangements, are linked to reduced substrate dependency, altered receptor distribution, and reprogramming of contact-mediated signaling pathways.

Collectively, µg induces profound reprogramming of tumor cell behavior by altering cytoskeletal architecture, disrupting mechanical equilibrium, and compromising the tensegrity framework. These findings not only deepen our understanding of cancer pathophysiology in space but also highlight mechanobiological vulnerabilities that could be exploited for targeted therapies on Earth.

### 4.2. Microgravity Induced Changes in Cell Proliferation and Cell Cycle Regulation

Microgravity, both in its real and s-µg forms, exerts profound effects on cancer cell proliferation and cell cycle regulation. Comprehensive reviews report that µg exposure across diverse tumor cell lines frequently leads to reduced proliferation, significant cell cycle arrest—commonly observed at the S and G2/M checkpoints—and the activation of apoptotic pathways. Additional studies note specific S/G2–M arrest and modulation of cyclins/CDKs) [134,140]. These outcomes are not solely due to the inhibition of growth signaling but are also linked to cytoskeletal reorganization and impaired cell–substrate adhesion.

In human glioma cells (U251), exposure to s-µg for three days led to growth inhibition, G2/M phase arrest, and apoptosis induction. These effects were attributed to the suppression of the FAK/RhoA/ROCK and FAK/Nek2 pathways, resulting in cytoskeletal instability and centrosome separation defects [141]. Concurrently, upregulation of *p21* and downregulation of *IGFBP-2*, both key regulators of cell survival, contributed to the impaired proliferation of U251 glioma cells [142]. These findings underscore the multifaceted impact of μg on cell cycle checkpoints, cytoskeletal integrity, and apoptotic regulation.

In FTC-133 cells, an in-flight study on the International Space Station (ISS) revealed that despite decreased *VEGFA* gene expression, there was a paradoxical increase in VEGF protein secretion [143].

In breast cancer models such as MCF-7 (breast cancer cell line) and MDA-MB-231, s-μg exposure resulted in marked proliferation inhibition, increased apoptosis, and S-phase accumulation [144]. Specifically, MDA-MB-231 cells exhibited elevated *Cyclin D3* and reduced *Bcl-2* expression, two key regulators of the G1/S checkpoint [145]. These findings align with broader evidence indicating that μg disrupts cell cycle orchestration, creating a “functional conflict” where cells initiate division but fail to complete it, ultimately shifting toward apoptosis—a mechanism that may be exploitable in anticancer therapy design.

A549 lung cancer cells exhibit increased multinucleation and growth suppression under s-μg, along with mitochondrial damage. These alterations are associated with dysregulation of key microRNAs—namely, upregulation of the pro-apoptotic miR-34a and downregulation of miR-21—highlighting a role for epigenetic modulation in cell cycle control and stress response under µg conditions [146]. Similarly, in the SCC cell line, s-μg exposure activates tumor suppressor pathways including TP53, CDKN2A, and PTEN, accompanied by enhanced apoptosis and cytoskeletal disorganization [147].

In the A673 (Ewing sarcoma cell line) cells, a 24 h s-μg exposure induced the formation of multicellular spheroids (MCS). This was accompanied by upregulation of *EWS/FLI1* and migration-related receptors such as *CXCR4* and *CD44*, particularly in the MCS subpopulation. While proliferation rates were not directly reported, these molecular changes suggest cell cycle remodeling and a potential shift toward a more aggressive phenotype [148].

Overall, the available evidence suggests that μg, through the inhibition of adhesion and growth pathways (e.g., FAK, RhoA, YAP1, Cyclin D), cytoskeletal remodeling, and modulation of apoptosis/survival regulators, exerts a time- and cell type-dependent suppressive effect on tumor proliferation. These observations emphasize the necessity of integrating tumor biology, exposure parameters, and µg platforms to unravel the therapeutic implications of μg in cancer treatment.

### 4.3. Regulation of Apoptosis Under Microgravity

µg, in both its real μg and s-µg forms, exerts multifaceted and profound effects on survival signaling, programmed cell death (apoptosis), and the expression of stress-responsive genes in cancer cells [26]. Numerous studies have demonstrated that μg can disrupt cell tensegrity, reorganize the cytoskeleton, suppress pro-survival pathways such as NF-κB, and activate key pro-apoptotic mediators including p53, Bax, FAS, and Caspase-8, ultimately impairing the viability and proliferative capacity of tumor cells [95,149].

In the MDA-MB-231 breast cancer cell line, a 7-day exposure to s-μg resulted in a marked increase in apoptosis, associated with reduced Bcl-2 expression (an anti-apoptotic protein), elevated secondary lysosomes, and the upregulation of cell death pathways [145]. These findings underscore the ability of μg-induced mechanical stress to override survival cues and initiate apoptotic programs. Similarly, in MCF-7 cells, increased expression of P53, PARP1, FAS, and Caspase-8 was observed in MCS, likely due to reduced adhesion and the consequent loss of integrin-mediated survival signaling [150].

The study conducted by Zhao et al. (2017) demonstrated that a 72 h exposure to s-µg significantly inhibited proliferation and induced apoptosis in U251 glioblastoma cells. These effects were associated with increased expression of *p21* and reduced expression of IGFBP-2, suggesting that the p21/IGFBP-2 axis may play a key role in mediating µg-induced cell death [142]. A recent study by Wang et al. (2025) demonstrated that ELKIN1, a mechanosensitive ion channel, is essential for mediating cellular adaptations to s-µg. ELKIN1 deletion inhibited microgravity-induced changes in cytoskeletal structure, focal adhesion remodeling, and YAP1 nuclear translocation, thereby preserving cellular integrity [151].

In SCC cells (squamous non-small cell lung cancer), s-μg exposure increased apoptosis in floating MCS populations, coinciding with severe actin cytoskeleton disruption and diminished adhesion [152]. Interestingly, pharmacologic inhibition of mTORC1 under 1g conditions mimicked μg-induced apoptosis, suggesting that suppression of this pathway may represent a shared mechanism [95,147]. Conversely, FAK/RhoA pathway activation reversed these effects, re-establishing cytoskeletal architecture and cell viability.

In A549 lung cancer cells, apoptosis was linked to a decrease in *Bcl-2* and alterations in stress-related miRNAs, notably an upregulation of *miR-34a* and downregulation of *miR-21*—pointing to an epigenetic component in μg-driven cell death [146]. Furthermore, in real µg (r-μg) and hyper-gravity (hyper-g) conditions, a paradoxical increase in BCL2 expression and activation of the VEGFA/EGF signaling axis have been reported, possibly reflecting a compensatory survival response to environmental stressors [153,154]. Collectively, strong evidence from in vitro models and spaceflight studies indicates that µg can systematically compromise tumor cell stability by inhibiting survival networks (NF-κB, Bcl-2, mTORC1), disrupting mechanical sensors (YAP1), impairing adhesion (FAK, RhoA), and triggering apoptosis via multiple converging pathways (p53, PARP1, p21, Bax). Crucially, these effects are more pronounced in malignant cells than in healthy ones, highlighting their heightened mechanosensitive and vulnerability under μg conditions. Understanding these mechanisms offers valuable insights not only for space biology but also for developing innovative cancer therapies that exploit biomechanical stress to overcome treatment resistance.

### 4.4. Tumor Migration, Invasion, and Metastasis Under Microgravity

µg has a significant impact on the aggressive behavior of cancer cells, including reduced migration, inhibition of matrix invasion, and modulation of metastatic potential [155]. These changes are mainly mediated by interference with cell adhesion-dependent pathways, cytoskeletal instability, and inhibition of cell mechanosensitive.

In U251 glioma cells, a 3-day exposure to s-µg markedly reduced migration and invasion capacity. This effect was associated with suppression of FAK, RhoA, and Nek2 kinase activity—pathways essential for actin cytoskeletal dynamics, lamellipodia formation, and directed cell motility under normal gravity. Inhibition of these signaling axes led to cellular arrest and significantly impaired matrix traversal [141].

In the MDA-MB-231 cell line, exposure to s-µg for 14 days caused a 35% reduction in migration and matrix invasion capacity. These effects were accompanied by a reduction in FAK phosphorylation and a decrease in RhoA activity, indicating inhibition of key mechano-transduction pathways. In addition, the position of the nucleus shifted from a polarized state to the center of the cell, indicating a disruption of polarity and directional movement ability [152,156].

S-µg significantly reduces migration in murine BL6-10 melanoma cells by more than 50%, coinciding with the suppression of FAK and RhoA activity. These changes highlight the crucial role of the FAK/RhoA axis in regulating melanoma cell migration under µg-like conditions [155].

From an epigenetic perspective, s-µg induces significant alterations in tumor cell miRNA content and exosome profiles. In FTC-133 cells, real µg exposure (e.g., during the CellBox-1 mission) led to significant modulation of over 100 miRNAs, many of which regulate invasion and metastasis [157,158]. Additionally, elevated surface levels of exosome CD63 and CD81—markers implicated in cell adhesion and motility—were observed, suggesting a shift toward altered exosome-mediated adhesion signaling in μg [158].

The role of the ELKIN1 protein as a key mechanosensor in the response of tumor cells to μg conditions has been identified. The lack of ELKIN1 prevents adhesion disruption but maintains the invasive phenotype of the cell. Conversely, its active presence leads to a reduction in invasion but also to an increase in damage to healthy cells under mechanical stress conditions [151].

In general, data show that µg, by inhibiting mechanical signals, reducing NF-κB phosphorylation, inhibiting FAK/RhoA, and weakening the nuclear localization of factors such as YAP1, leads to a reduction in the invasion, migration, and potential metastasis of cancer cells. However, the responses are highly dependent on cell type, the level of expression of mechanosensory, and the duration of exposure. Therefore, to translate these findings into clinical treatments, in vivo studies and multicellular models are needed.

A summary of key studies examining immune and cancer-related responses under real and simulated µg conditions is presented in Table 1.

## 5. Therapeutic Implications of Microgravity: Drug Resistance and Immunotherapy Perspectives

Growing evidence suggests that µg can profoundly alter the responsiveness of cancer cells to drug treatments. In two-dimensional (2D) cell culture models, exposure to µg has been associated with increased programmed cell death (apoptosis) and a reduction in cellular metabolic defenses, thereby enhancing the sensitivity of cells to chemotherapeutic agents. In contrast, studies using three-dimensional (3D) models such as spheroids—which more closely mimic the architecture and microenvironment of in vivo tumors—have reported enhanced drug resistance. These divergent outcomes are likely driven by µg-induced alterations in oxidative phosphorylation pathways, cell cycle regulation, and apoptosis control mechanisms, highlighting the complex nature of tumor responses under weightless conditions.

A representative study by Takahiro Fukazawa and colleagues investigated the effects of s-µg on A549 cells. They found that µg increased the sensitivity of these cells to cisplatin, primarily via early activation of the caspase-3-dependent apoptotic cascade and engagement of p53-independent cell death mechanisms, such as autophagy. These findings underscore the capacity of µg to reprogram cell death signaling in non-canonical ways [159].

In the context of hematological malignancies, the K562 cell line has been widely studied [160]. One experiment examined the effects of daunorubicin and doxorubicin following 48 h of µg exposure [160]. Interestingly, daunorubicin led to increased cell migration under µg conditions—suggesting a possible shift in cellular behavior and drug responsiveness—while doxorubicin induced enhanced migration in both µg and normal gravity, indicating a drug-specific interaction with the gravitational context.

In contrast, a separate study using the same K562 line treated cells with hydroxyurea and paclitaxel and reported reduced drug efficacy under µg [161]. Notably, the nucleus-to-cytoplasm ratio—an indicator typically reduced in response to hydroxyurea—remained unchanged under µg [161]. This suggests that µg may contribute to drug resistance in certain contexts by attenuating pathways involved in cell cycle arrest or apoptosis. Although these studies provide compelling evidence that µg modulates drug responsiveness in cancer cells, the observed heterogeneity—arising from differences in cancer cell types (e.g., A549 vs. K562), drug classes (alkylating agents, antitumor antibiotics, mitotic inhibitors), and the parameters of µg exposure—indicates that therapeutic responses in space are highly context-dependent.

Beyond cytotoxic agents, µg may profoundly affect the efficacy of immunotherapy. Immunotherapeutic modalities—including checkpoint inhibitors (PD-1/PD-L1, CTLA-4), CAR-T and CAR-NK cell therapies, cytokine-based treatments (e.g., IL-2, TNF-α), cancer vaccines, and oncolytic viruses—are all susceptible to immune remodeling induced by µg. For instance, murine models onboard the ISS vaccinated with tetanus toxoid and CpG exhibited cardiac inflammation accompanied by NF-κB activation and elevated levels of IFN-γ, IL-6, and IL-17, indicating altered vaccine-induced immune responses [162].

Preclinical studies have also shown that µg may enhance cancer vaccine efficacy. Mesenchymal stem cells (MSCs) cultured under s-µg exhibited potent antitumor properties, including increased MHC-I and heat shock protein expression, the stimulation of CD8^+^ T cell and Th1 responses, and a reduction in Treg populations [163]. Given their low immunogenicity, MSCs are considered promising vectors for space-adapted immunotherapies. Moreover, A549 lung cancer cells exposed to µg regained epithelial characteristics—such as elevated E-cadherin and reduced N-cadherin—alongside upregulation of humoral immunity-related genes including *FCGBP*, *BPIFB*, and *CST1*, which may serve as immunomodulatory biomarkers or therapeutic targets in µg-associated oncology [164].

A compelling feature of μg conditions is their potential to reverse tumor-induced immune evasion. In murine lymphoma models, cancer cells that previously inhibited DC activation and T cell priming lost their immunosuppressive function following s-μg exposure [65]. This change resulted in elevated IL-2 and IFN-γ secretion and increased CD4^+^ and CD8^+^ T cell cytotoxicity [65]. Notably, injecting these “reprogrammed” tumor cells into mice led to reduced tumor growth, suggesting enhanced immunogenicity under µg.

Furthermore, studies on SH-SY5Y neuroblastoma cells infected with HSV-1 under s-μg revealed decreased viral replication but increased pro-inflammatory cytokines (IL-1β, IL-6, TNF-α) and HERV activation, pointing toward immune dysregulation inflammation [28]. Future studies are needed to evaluate drug efficacy and immunotherapy performance under spaceflight conditions.

## 6. Translational Considerations and Future Directions

### 6.1. Translational Relevance and Clinical Implications

One of the most significant contributions of µg (µg) research to cancer biology is the ability to generate physiologically relevant three-dimensional (3D) tumor models without the need for artificial scaffolds [13,165]. Multiple cancer types—including thyroid carcinoma (TC), breast cancer (BC), colorectal cancer (CRC), hepatocellular carcinoma (HCC), lung cancer (LC), and prostate cancer (PC)—have been shown to form multicellular spheroids (MCS) under both real and simulated µg conditions [144,165]. These spheroids, and in some cases organoids, replicate key hallmarks of in vivo tumors such as cell–cell and cell–matrix interactions, oxygen and nutrient gradients, and intratumoral heterogeneity, which are largely absent in conventional two-dimensional cultures [13,165]. Such models provide advanced platforms for studying cancer progression, angiogenesis, tumor–stroma interactions, and immune cell infiltration, while improving the predictive value of preclinical drug screening compared to standard monolayer systems.

Although µg is not a therapeutic modality itself, it serves as a unique mechanistic amplifier that reveals mechano-sensitive signaling pathways that are often masked under normal gravity. Pathways involving NF-κB, FAK/RhoA, ERK/RELA, PI3K/AKT, and YAP/TAZ undergo significant modulation under µg, leading to cytoskeletal reorganization, focal adhesion disassembly, and transcriptional reprogramming that collectively influence epithelial–mesenchymal transition (EMT), apoptosis, and metastatic potential. Notably, NF-κB suppression—frequently reported in immune and stromal cells under µg—emerges as a critical regulatory node, as its inhibition reduces transcription of pro-inflammatory cytokines and costimulatory molecules, thereby weakening antitumor immunity and shaping a tumor-permissive microenvironment. Notably, multi-omics studies from the NASA Twins Study and SOMA program confirm that µg induces transcriptional and epigenetic reprogramming in immune and stromal cells, including promoter hypermethylation in NOTCH3 and SLC1A5, persistent inflammatory cytokine elevations (IL-6, TNF-α, VEGF), FOXP3 upregulation, and long-term MHC-I suppression [51,52].

These mechanistic insights are relevant to terrestrial oncology because they identify vulnerabilities that drive drug resistance and immune evasion in aggressive tumors. Targeting such mechano-regulated pathways, for instance through pharmacological inhibition of FAK or YAP, has already demonstrated therapeutic potential in preclinical cancer models, underscoring the translational value of µg research for the development of anti-metastatic strategies. Combining these mechanistic insights with advanced immunotherapies under µg-driven 3D models may uncover novel resistance pathways and synergistic treatment strategies. Importantly, while no studies have yet directly assessed immune checkpoint inhibitors (e.g., PD-1/PD-L1, CTLA-4) or CAR-T/CAR-NK therapies under real or simulated µg, this represents a key gap. Indirect evidence—such as altered adaptive immune responses during vaccination on the ISS and µg-induced remodeling of T-cell and NK-cell signaling—underscores the potential vulnerability of immunotherapies to gravity-dependent immune modulation [163]. Furthermore, HERV-derived peptides and envelope proteins have emerged as promising immunogenic targets for CAR-T therapies and cancer vaccines, supported by encouraging preclinical results [119,120,121].

Importantly, these findings are not limited to theoretical considerations. NF-κB suppression under µg conditions parallels mechanisms of immune escape and therapeutic resistance observed in aggressive Earth-based tumors, suggesting that pharmacological strategies to restore NF-κB activity could synergize with immune checkpoint blockade [166]. Similarly, HERV reactivation under µg highlights the potential for leveraging epigenetic therapies to induce viral mimicry and enhance tumor immunogenicity—an approach already under clinical evaluation in combination with PD-1 inhibitors [167]. These mechanistic overlaps bridge µbiology with actionable therapeutic strategies, positioning µg-driven models as practical platforms for identifying resistance mechanisms, refining CAR-T or CAR-NK protocols, and designing combination regimens tailored to immune and epigenetic vulnerabilities.

Future translational efforts should focus on integrated µg-driven platforms combining patient-derived tumor organoids, immune co-cultures, and advanced multi-omics profiling to systematically evaluate immunotherapy performance. Conceptually, these systems can also facilitate identification of resistance mechanisms and inform novel combination strategies, including epigenetic therapy paired with checkpoint blockade.

Given the recent demonstration of widespread immune and epigenetic remodeling in astronauts [48,51,52], incorporating these human-derived signatures into µg-based models will improve clinical relevance and help close the current gap between in vitro findings and patient-specific applications.

### 6.2. Future Directions

Although significant progress has been made, critical gaps remain in translating microgravity (µg) research into practical applications for cancer therapy. Most current studies investigate tumor and immune responses independently; however, future research should integrate these components using advanced models. Co-culture platforms integrating patient-derived tumor organoids with immune cells, along with organ-on-chip technologies, are essential for accurately modeling the tumor–immune microenvironment under µg conditions [168,169,170]. While similar strategies have been successfully applied in terrestrial oncology to study immune–tumor interactions and therapeutic responses [171,172], no studies have yet implemented such integrated models in µg, underscoring a critical gap that future research must address.

Another challenge is the variability in experimental outcomes due to differences in cell types, µg simulation platforms, and exposure durations. To address this, standardized protocols and comprehensive multi-omics approaches—including transcriptomics, proteomics, and epigenomics—are needed to identify reproducible molecular signatures. Incorporating automated bioreactor systems and high-throughput screening could further enhance reproducibility and enable the large-scale evaluation of anticancer drugs and immunotherapies in µg-adapted platforms.

Understanding how µg influences therapeutic efficacy is a key priority. While no studies have yet tested immune checkpoint inhibitors (e.g., PD-1/PD-L1, CTLA-4) or CAR-based therapies in µg, systematic evaluation is essential to predict efficacy shifts or resistance mechanisms. A feasible near-term strategy involves in vitro functional assays combining CAR-T or CAR-NK cells with tumor spheroids under simulated µg to assess cytotoxicity, exhaustion markers, and checkpoint expression, followed by omics-based profiling of immune pathways. These efforts can inform the design of later in vivo experiments using humanized or murine models in spaceflight environments.

Processes such as HERV activation and related inflammatory pathways should also be investigated, as they may contribute to immune dysfunction and tumor progression under µg. HERV expression patterns could serve as biomarkers for immune dysregulation and therapeutic targets in both spaceflight and terrestrial oncology.

Finally, for long-duration space missions, combined countermeasures—such as partial gravity through centrifugation and pharmacological or immunological interventions—may be required to maintain immune competence and reduce cancer risk. Addressing these areas will not only safeguard astronaut health but also accelerate the translation of µg-based insights into precision cancer therapies on Earth.

## Figures and Tables

**Table 1:** Summary of Immune and Cancer Studies under Real and Simulated Microgravity.

Cell Type/System	µg Platform	Exposure Time	Key Observed Effect	Reference
Human (astronaut skin biopsies, *n* = 4)	Real µg	Short (3-day flight; pre- and 1-day postflight)	↑ KRAS and inflammatory pathways across skin layers; altered interferon response, DNA damage, epithelial barrier disruption, T-cell migration	[48]
T cells (primary and Jurkat); Monocytes/macrophages (U937, etc.)	Simulated µg (2D clinostat), real µg (parabolic flight)	Short-term (minutes–hours)	Jurkat: ↑ ERK/MEK/p38, ↓ NF-κB nuclear translocation; U937: c-Jun activation ↑ or ↓ (stim-dependent); ↑ p53 phosphorylation	[43]
Mouse macrophage (RAW 264.7 cell line)	Simulated µg	8, 24, 48 h	↓ viability, ↓ phagocytosis, shift in DEGs (↑ at 8–24 h, ↓ at 48 h), cytoskeleton disruption (Arp2/3)	[62]
Human B lymphoblasts (HMy2.CIR cells)	Simulated µg	30 min	↑ apoptosis under heavy ion radiation, ↑ ROS, ↑ ERK/MKP-1/caspase-3 activation	[81]
Human astronauts (PBMC)	Real µg	135–210 days	↓ mitochondrial function (OXPHOS), ↑ T cell exhaustion markers (CTLA-4, TIGIT, PD-L1, EOMES), ↓ calcium/GPCR signaling	[78]
human T cells	Real µg	1.5 h	↓ Rel/NF-κB, CREB, SRF activation; ↓ cREL; inhibited TNF pathway → impaired early T cell activation	[83]
Human cells (Jurkat), monocytes (U937)	Real µg	Seconds to minutes	↓ HIF-1α expression (mRNA and protein), ↓ PDK1 transiently; adaptation within ~5 min	[85]
Human astraunautPBMCs	Real µg	~3 days	↑ FOXP3^+^ Tregs; ↓ MHC I genes; ↑ inflammatory cytokines	[17]
Human (in vitro PBMCs) + validation with human/mouse in vivo	simulated µg + validation with real µg (I4, ISS, JAXA)	25 h	↓ T/NK cytotoxicity; ↑ monocyte inflammation; dysregulated IFN and IL-6; cytoskeletal changes; pyroptosis; sirtuin modulation	[27]
Human astronauts Plasma	Real µg	9–14 days	↑ IL-1α, IL-6, IL-8, IFN-γ, IL-4, IL-10, IL-12, IL-13, eotaxin, IP-10; strong Th2 shift (IL-4 ↑ 21×)	[50]
THP-1-derived macrophages (M0, M1, M2 phenotypes)	Simulated µg	48 h	↓ TNF-α; ↑ IL-12 and VEGF (all phenotypes); ↑ IL-10 (M1 and M2)	[44]
Natural Killer (NK) cells	Simulated µg	48 h	↓ NK cytotoxicity vs. K-562/MOLT-4; ↓ GZMB, PRF1; ↑ impairment with prolonged exposure	[54]
Human astronauts	Real µg	180–360 day	↓ NK cytotoxicity (~50%) vs. K-562; no change in NK count/receptors; greater decline in rookies	[58]
Human PBMCs (in vitro)	Simulated µg	18 h	↓ NK, CD4^+^, CD8^+^ function; ↑ Treg function (STAT5 activation)	[55]
Primary mouse macrophages	Simulated µg	24 h	↑ Arginase-1 (M2); ↑ IL-6; ↓ IL-12B (p40); p38 MAPK → C/EBPβ pathway	[67]
Human thyroid cancer (FTC-133 cells)	Real µg	~6.5 min	↑ cytoskeletal gene expression, disruption of F-actin bundles (holes, loss of filopodia/lamellipodia), ↑ ACTB, EZR, RDX, MSN; ↓ SEPT11 → altered cytoskeleton dynamics	[134]
Human breast epithelial (MCF 10A, LINC-intact vs. disrupted)	Simulated µg	2–20 h	SMG-induced nuclear shape change requires LINC; LINC-dependent gene expression (e.g., ↑ RBMS3, altered ECM/adhesion genes)	[136]
Human Ewing’s sarcoma (A673)	Simulated µg	24 h	↑ EWS/FLI1 in both adherent and spheroids; ↑ CXCR4 and CD44 in spheroids; ↑ CAV1; ↓ DKK2, ↓ VEGF-A under s-µg	[148]
Mouse melanoma (B16 BL6-10)	Simulated µg	24 h	↑ apoptosis; ↓ focal adhesions and cytoskeleton stability; suppression of FAK/RhoA → ↓ mTORC1/NF-κB and ERK1/2 signaling; ↓ NEPs (lamin-A, emerin, sun1, nesprin-3)	[95]
Human breast cancer (MCF-7)	Simulated µg	24 h	↑ multicellular spheroid formation; NFκB p65 translocates to nucleus; ↑ HMOX1, ANXA1, ANXA2, CTGF, CAV2, ICAM1, FAS, Casp8, BAX, p53, CYC1, PARP1	[150]
Human glioma (U251MG)	Simulated µg	smg	↑ apoptosis (↑ p21, ↓ IGFBP-2), ↓ proliferation	[142]
Human lung cancer (squamous)	Simulated µg	Up to 96 h	↑ 3D spheroid formation → ↑ apoptosis (spheroids), ↑ TP53, CDKN2A (p14), RB1, PTEN; actin shifts to spherical alignment; SOX2 ↑ in adherent cells	[147]

Legend: Arrows indicate direction of molecular changes and pathway activation. ↑ = upregulation; ↓ = downregulation; → = activation of downstream pathway.
